# Synergistic Viral Replication of Marek’s Disease Virus and Avian Leukosis Virus Subgroup J is Responsible for the Enhanced Pathogenicity in the Superinfection of Chickens

**DOI:** 10.3390/v10050271

**Published:** 2018-05-18

**Authors:** Jing Zhou, Guo-Liang Zhao, Xiao-Man Wang, Xu-Sheng Du, Shuai Su, Chen-Gui Li, Venugopal Nair, Yong-Xiu Yao, Zi-Qiang Cheng

**Affiliations:** 1 College of Veterinary Medicine, Shandong Agricultural University, Tai’an 271018, China; tiramisurd@163.com (J.Z.); 13515381026@163.com (G.-L.Z.); m15253197976@163.com (X.-M.W.); duxusheng12345@163.com (X.-S.D.); ssu6307@163.com (S.S.); chgli1981@126.com (C.-G.L.); 2 Shandong Provincial Key Laboratory of Animal Biotechnology and Disease Control and Prevention, Tai’an 271018, China; 3 The Pirbright Institute & UK-China Centre of Excellence on Avian Disease Research, Pirbright, Ash Road, Guildford, Surrey GU24 0NF, UK; venugopal.nair@pirbright.ac.uk (V.N.); yongxiu.yao@pirbright.ac.uk (Y.-X.Y.)

**Keywords:** Marek’s diseases virus, avian leukosis virus subgroup J, superinfection, synergism, pathogenicity

## Abstract

Superinfection of Marek’s disease virus (MDV) and avian leukosis virus subgroup J (ALV-J) causes lethal neoplasia and death in chickens. However, whether there is synergism between the two viruses in viral replication and pathogenicity has remained elusive. In this study, we found that the superinfection of MDV and ALV-J increased the viral replication of the two viruses in RNA and protein level, and synergistically promoted the expression of IL-10, IL-6, and TGF-β in chicken embryo fibroblasts (CEF). Moreover, MDV and ALV-J protein expression in dual-infected cells detected by confocal laser scanning microscope appeared earlier in the cytoplasm and the nucleus, and caused more severe cytopathy than single infection, suggesting that synergistically increased MDV and ALV-J viral-protein biosynthesis is responsible for the severe cytopathy. In vivo, compared to the single virus infected chickens, the mortality and tumor formation rates increased significantly in MDV and ALV-J dual-infected chickens. Viral loads of MDV and ALV-J in tissues of dual-infected chickens were significantly higher than those of single-infected chickens. Histopathology observation showed that more severe inflammation and tumor cells metastases were present in dual-infected chickens. In the present study, we concluded that synergistic viral replication of MDV and ALV-J is responsible for the enhanced pathogenicity in superinfection of chickens.

## 1. Introduction

Marek’s disease (MD), caused by *gallid herpesvirus 2* (GaHV-2) also known as MD virus (MDV), is a highly infectious lymphoproliferative disease of chickens. MDV is a member of the *Mardivirus* genus in the subfamily of *Alphaherpesvirinae* [1]. Susceptible chickens develop visceral and neural lymphomatous lesions [2,3], resulting in death or carcass condemnation. As a complex disease, MD is characterized by neurological signs, immunosuppression, and neoplastic transformation of T lymphocytes, localized around peripheral nerves and the visceral organs of the host [4,5].

Cell-associated live avirulent and nononcogenic vaccine strains have been successfully used to immunize chickens against MD since the 1970s [6]. However, vaccination does not prevent superinfection and the shedding of virulent challenge viruses [7,8], thus, chickens can potentially be infected simultaneously with both vaccine and virulent MDV strains. In addition, the virulence of MDV-1 has persistently increased possibly because of highly intensive farming [9], imperfect vaccinations [10,11], or mixed infection with other pathogens, such as avian leukosis virus subgroup J (ALV-J) [12,13], reticuloendotheliosis virus (REV) [14], avian hepatitis E virus (AHEV) [15], chicken anemia virus (CAV) [16], and so on. The increased virulence of MDV-1 promoted its tumorigenesis and pathogenicity.

Avian leukosis virus subgroup J, an oncogenic retrovirus of poultry, was first isolated by Payne in 1991 in the UK [17]. ALV-J mainly causes myelocytomas and other tumors, including hemangiomas, lymphomas, fibrosarcomas, histiocytic sarcomas, and induces subclinical symptoms, including immunnosuppression, weight loss, and a drop in egg production, leading to great economical losses in the poultry industry [18,19,20,21]. A remarkable property of ALV-J is its genetic diversity and instability, resulting from high sequence mutation rates, particularly of the viral gp85 glycoprotein, which is responsible for viral immune evasion and persistence [21].

Generally, the term “coinfection” is used to describe simultaneous infection of cells or animals with two viruses, “superinfection” to describe infection of cells or animals with two viruses at different times, and “dual infection” to describe infection of individual cells or animals with two viruses. Superinfection of MDV and ALV-J occurs commonly in nature [22,23], and causes more severe immunosuppression, pathogenicity, and extended tumor spectrum [12]. Synergistic superinfection of MDV and ALV-J is a great threat to the poultry industry, thus, it is important to reveal the underlying mechanisms of the enhanced pathogenicity.

## 2. Materials and Methods

### 2.1. Viruses, Cells, and Animals

The animal experiments described in this study were approved by the Shandong Animal Care and Use Committee (SDAU number 17-095, 5 September 2017). Serotype 1 MDV strains, Md5 (very virulent, vv) at passage 12 in chicken embryo fibroblast (CEF) were used as MDV infection. The titer of MDV was determined by plaque assay. The NX0101 strain of ALV-J was maintained in our laboratory. The 50% tissue culture infectious dose (TCID_50_) of NX0101 strain was titrated by limiting dilution in DF-1 cell line culture. CEF were maintained in Dulbecco’s Modified Eagle’s Medium (DMEM) supplemented with 10% fetal bovine serum (FBS), 1% l-glutamine, 1% penicillin/streptomycin, and in a 5% CO_2_ incubator at 37 °C. Day old Leghorn specific-pathogen-free (SPF) chickens (Saisi) were maintained under SPF conditions.

### 2.2. Quantitative Reverse Transcription PCR (RT-qPCR)

Total RNA was extracted from CEFs or tissues, and then reverse transcribed to cDNA using the Taqman Gold Reverse Transcription kit (Applied Biosystems, Shanghai, China). The 20 µL reaction contained 1 µL cDNA, 0.4 µL Rox Reference Dye II (50×), 10 µL SYBR Premix Ex TaqTM (TaKaRa, Shanghai, China), and 8 pM primers for ALV-J and MDV as is shown in Table 1. The GAPDH mRNA level was used as the internal control. The reactions were run on an Applied Biosystems 7500 Prism real-time PCR machine with the following program: (1) 95 °C 30 s, 1 cycle; (2) 95 °C 5 s, 60 °C 34 s, 40 cycles. The 2^−∆∆*C*t^ method was used to analyze the results.

### 2.3. Western Blot

Total protein lysates were isolated from treated CEF cells using a lysis buffer (pH 7.6, 0.1 mmoL/L NaCl, 0.01 mmoL/L Tris-HCl, 0.001 moL/L Ethylene diamine tetraacetic acid (EDTA), pH 8.0, 1 μg/mL Aprotinin, 100 μg/mL PMSF). The protein concentrations were measured by BCA Protein Assay Kit (PIERCE, Rockford, IL, USA). The proteins were separated by 10% SDS-PAGE, transferred to polyvinylidene fluoride membranes, which were blocked for 2 h in 5% defatted milk in Tris-buffered saline containing Tween-20 (10 mM Tris-HCl, 150 mM NaCl, tris-buffered saline (TBS), 0.1% Tween-20). The ALV-J SU protein was detected using the 1D4 monoclonal antibody (dilution, 1:1000). The MDV Meq protein was detected using the BA4 monoclonal antibody (dilution, 1:1000). Glyceraldehyde-3-phosphate dehydrogenase (GAPDH) was used as loading control (Ab dilution 1:4000; predicted molecular weight: 42 kDa). The blots were then developed by incubation in a chemiluminescence substrate and exposed to X-ray films.

### 2.4. Confocal Laser Scanning Microscope Assay

**Figure 1:**
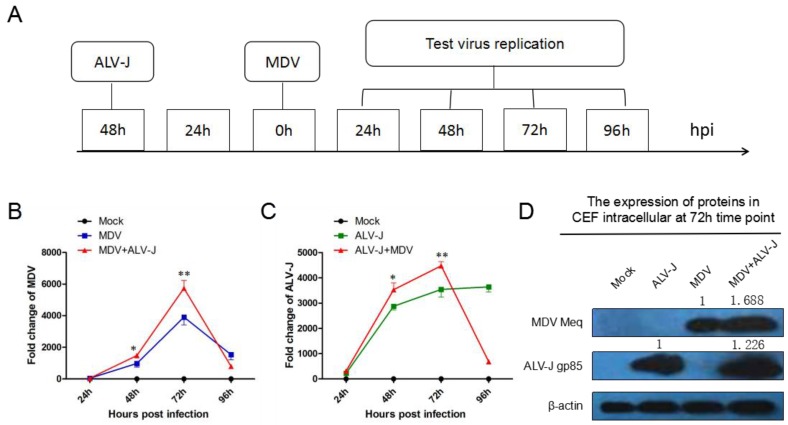
Time course of superinfection of viral replication tested by RT-qPCR and western blot. (**A**) Time course of superinfection of avian leukosis virus subgroup J (ALV-J) and Marek’s disease virus (MDV). (**B**) RNA level of MDV *Meq* gene from 24 hpi to 96 hpi. (**C**) RNA level of ALV-J env gene from 24 hpi to 96 hpi. (**D**) Protein expression level of MDV Meq and ALV-J gp85 at 72 hpi.* = significant difference; ** = extremely significant difference.

CEF cultures were infected with ALV-J, MDV, and MDV+ALV-J on sterile glass coverslips in 24-well dishes at 10^3.8^ TCID_50_ of ALV-J or 50 PFU of MDV per well. According to the designated time course of superinfection as shown in Figure 1A, the CEF cultures were fixed with ice-cold 40% ethanol and 60% acetone for 7 min and then washed twice with phosphate-buffered saline (PBS). The CEF cultures used for viral protein detection were blocked in 10% neonatal calf serum, and then stained with monoclonal antibody BA4 (dilution, 1:1000) against the MDV protein Meq or monoclonal antibody 1D4 (dilution, 1:1000) against the ALV-J protein SU at 37 °C for 1 h. FITC-labeled goat anti-mouse IgG (for MDV) and PE-labeled goat anti-mouse IgG (for ALV-J) were used as secondary antibody incubating at 37 °C for 1 h. The overlapping of the two colors of fluorescent markers appears yellow. MDV+ALV-J infected cells were tested by the two colors of fluorescent labeled secondary antibody. The nuclei of all the infected cells were stained by 4′,6-diamidino-2-phenylindole (DAPI). Finally, the cells were covered with 50% glycerin and examined under laser confocal microscopy (Leica SP8, Berlin, German).

### 2.5. ELISA for Cytokines

A total of 5 mL supernatant was collected from cultured CEFs, and then centrifuged at 425× *g* for 20 min. To measure the concentrations of IL-6, IL-10, TGF-β in the cell culture supernatant, ELISA tests were carried out using commercially available kits from Senbeijjia Biological Technology. The preparation of all reagents, the working standards, and protocol were followed according to the manufacturer’s instructions.

### 2.6. Pathogenicity Assay in SPF Chickens

SPF chicks were divided into four groups (*n* = 20 per group) and placed in separate isolators receiving filtered positive-pressure air. At 1 day of age, chicks in the ALV-J and MDV+ALV-J groups were inoculated into the abdominal cavity with ALV-J of 100 μL 10^3.8^ TCID_50_, and at 3 days of age, chicks in the MDV and MDV+ALV-J groups were inoculated via the abdominal cavity with MDV at a dose of 2000 plaque-forming units (PFUs) in 100 µL volume of dedicated diluents. Chicks in the Mock group were inoculated with 100 μL DMEM at 1 day of age. Clinical performance and mortality were recorded each week. All chickens were euthanatized and examined post-mortem at 10 weeks. Tissues from each chicken were divided into two portions for histopathology and qPCR assay.

### 2.7. Histopathological Examination

The tissues collected at necropsy were fixed in 10% formaldehyde, for the standard processing, and embedded in paraffin wax. 4 µm tissue sections were examined after hematoxylin and eosin staining.

## 3. Results

### 3.1. MDV and ALV-J Synergistically Increase Viral Replication In Vitro

To understand the synergistic viral replication of MDV and ALV-J, CEF cultures were inoculated with phosphate buffer (Mock), MDV, ALV-J, and both viruses (MDV+ALV-J), respectively. The time course of superinfection is shown in Figure 1A. Total RNA was extracted from the infected cells for RT-qPCR analyses at 24, 48, 72, and 96 hours postinfection (hpi). The accumulation level of MDV RNA was significantly higher in dual-infected cells than that in single-infected cell at 72 hpi (1.47-fold) as shown in Figure 1B, while the ALV-J RNA was significantly increased at 48 (1.21-fold) to 72 hpi (1.26-flod) as shown in Figure 1C. However, after 72 hpi, the RNA level of the two viruses in the dual-infected cells suddenly decreased to a very low level due to the accelerated cell death. Next, the expression levels of MDV and ALV-J were quantified by Western blotting at 72 hpi. The results showed that ALV-J increased MDV protein expression 1.688-fold and MDV promoted ALV-J protein expression 1.226-fold (Figure 1D), in agreement with the RNA level. Taken together, dual-infection of MDV and ALV-J synergistically increased the expression levels of both the viral gene transcript and protein.

### 3.2. MDV and ALV-J Synergistically Induce Cytopathy

**Figure 2:**
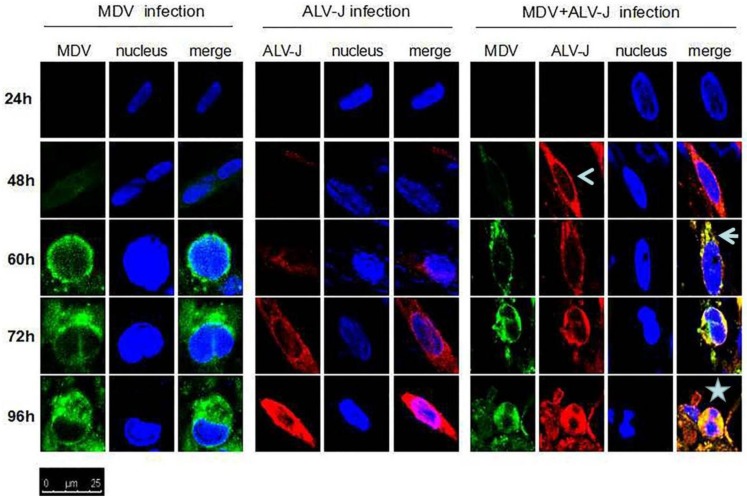
Protein expression and localization of MDV and ALV-J examined by confocal laser scanning microscope (LSCM). Surface protein (SU) of ALV-J in superinfected cells was expressed earlier (open arrowhead) than those of single-infected cells. The proteins of ALV-J and MDV were co-localized in cytoplasm (arrowhead). The dual-infected cells showed more cytopathy (CPE) (star) than single-infected cells. Fluorescein isothiocyanate (FITC)-labeled goat anti-mouse IgG (for MDV) (green) and P-phycoerythrin (PE)-labeled goat anti-mouse IgG (for ALV-J) (red) were used as the secondary antibody in the assay.

Cytopathic effects (CPE) in MDV and MDV+ALV-J-infected CEFs were observed and the specificity was confirmed by confocal laser scanning microscopy (CLSM) using MDV Meq-specific and ALV-J gp85-specific monoclonal antibodies. The CLSM assay showed that the fluorescent signal and the CPE in dual-infected cells appeared 24 h earlier than those of single-infected cells (Figure 2). The merged signal in dual-infected cells indicated that the expression of the two proteins of Meq and SU might utilize a similar pathways for the biosynthesis of viral-proteins. The data throws light on the possible synergistic mechanism of MDV and ALV-J.

### 3.3. Inflammatory Mediator Secretion

**Figure 3:**
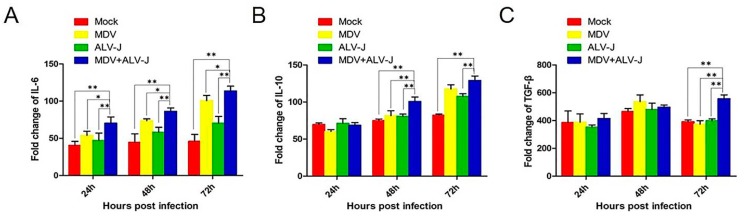
Detection of inflammatory mediator of IL-6, IL-10, and TGF-β by ELISA. (**A**) IL-6 secretion in dual-infected cells showed a significant increase (*p* < 0.05) from 24 h postinfection (hpi) to 72 hpi. (**B**) IL-10 secretion in dual-infected cells showed a significant increase (*p* < 0.01) from 48 hpi to 72 hpi. (**C**) TGF-β secretion in dual-infected cells showed a significant increase (*p* < 0.01) at 72 hpi. ** = extremely significant difference.

Several inflammatory mediators, such as, IL-6, IL-10, and TGF-β, have been shown to be involved in both the initiation and progression of cancer. To understand if the two viruses synergistically promote inflammatory mediator secretion, we tested the dynamic changes of IL-6, IL-10, and TGF-β expression by ELISA [24]. The results showed that the superinfected cells were found to have significantly higher levels of IL-6 (*p* < 0.05) (from 24 to 72 hpi), IL-10 (*p* < 0.01) (from 48 to 72 hpi) and TGF-β (*p* < 0.01) (at 72 hpi) compared to the controls (Figure 3).

### 3.4. Mortality and Survival Curve

**Figure 4:**
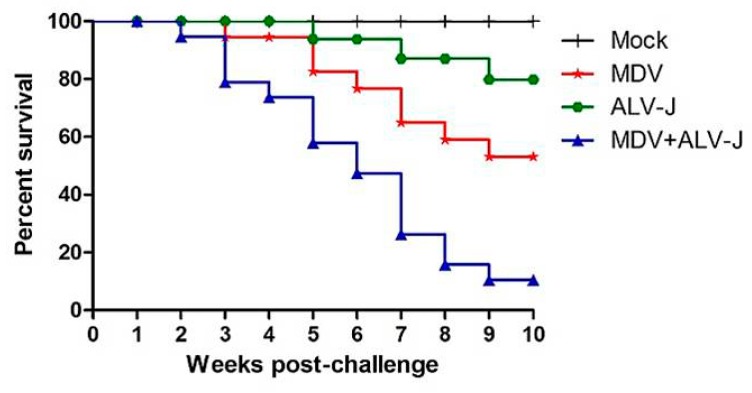
Survival curves for each group. Comparison of survival curves between the Mock, ALV-J, MDV, and MDV+ALV-J group.

During the in vivo infection, all chickens in the MDV-infected, and the MDV+ALV-J dual-infected groups developed MD, and no chickens in the Mock group or ALV-J-infected group were MD positive. As shown in Figure 4, the overall mortality of MDV-, ALV-J-infected chickens was 20% and 5%, respectively, while that of the MDV and ALV-J dual-infected group was 45%. No chickens showed mortality in the Mock-infected group. These results suggested that the severity of disease caused by dual-infection was more serious than with MDV or ALV-J infection alone. The survival period of chickens in the MDV and ALV-J dual-infected group was generally shorter than that of diseased chickens in the MDV or ALV-J single-infected group, as shown by the survival curves shown in Figure 4.

### 3.5. Tumorigenesis and Pathogenicity In Vivo

The presence of tumors was determined by gross examinations in the visceral organs of infected chickens. MDV infection induced tumor development in 60% chickens. These tumors were most commonly found in the liver and proventriculus, followed by the heart, kidneys, and spleen. In contrast, the tumor formation rate of MDV+ALV-J dual-infected chickens was much higher (90.0%) than that of chickens infected with MDV alone. MDV+ALV-J dual-infected chickens most commonly exhibited tumors in the liver and kidneys, followed by the proventriculus and spleen. These results indicated that MDV and ALV-J dual-infection significantly increased the rate of tumor development and changed the distribution of visible tumors in chickens, compared to those infected with MDV alone. No visible tumorigenesis was observed in the ALV-J-infected group or in the Mock group during the experimental period.

**Figure 5:**
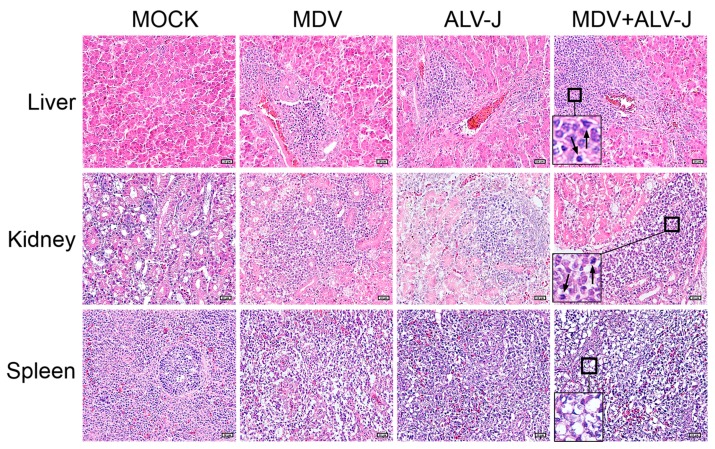
Histological lesions of each group (hematoxylin-eosin, H.E 400×). In dual-infected livers and kidneys, more severe pathogenicity and tumor cells metastases (arrow) were observed. In the dual-infected spleens, more severe lymphocytic depletion (square) was observed.

The histological results are shown in Figure 5. As shown, the invasion and proliferation of neoplastic lymphoid cells in the tissues of MDV+ALV-J dual-infected chickens were generally more severe than those in the tissues of MDV-infected chickens. The spleen in dual-infected group showed more severe lymphocytic depletion than that in single infected group. No histological lesions were observed with chickens in the Mock-infected group, and slight lymphocytic infiltrates were observed in the liver, spleen, kidneys, and proventriculus of chickens in the ALV-J-infected group. No myelocytomas were observed in the ALV-J infected or dual-infected group.

### 3.6. Virus Load in Tissues

**Figure 6:**
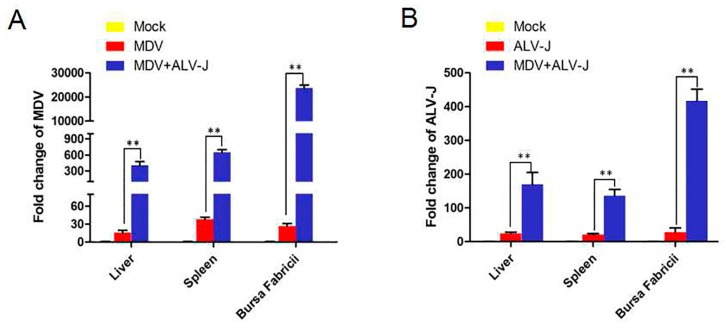
Viral loads in tissues. (**A**) MDV loads in liver, spleen, and *Bursa Fabricii*. (**B**) ALV-J loads in liver, spleen, and *Bursa Fabricii*. ** = extremely significant difference.

To analyze viral copies of MDV and ALV-J in tissues of infected chickens, the liver, spleen, and *Bursa Fabricii* were tested by qPCR at 21 dpi. The results showed that the viral load of MDV in the liver, spleen, and *Bursa Fabricii* of MDV+ALV-J-infected chickens increased 20.69-fold, 16.96-fold, and 889.61-fold, respectively, far more than in single MDV infected chickens (Figure 6A); while ALV-J increased 6.97-fold, 6.39-fold, and 14.9-fold, respectively (Figure 6B). Interestingly, the synergistic replication of ALV-J and MDV in dual-infected chickens is much higher than those in dual-infected CEFs. The results suggested that ALV-J infection has a discernable effect on the replication of MDV in vivo.

## 4. Discussions

MD is the first tumor disease that can be controlled by vaccine. Nononcogenic turkey herpesvirus (HVT), nononcogenic chicken herpesvirus 3 (gallid herpesvirus 3, GaHV-3), and attenuated MDV (attGaHV-2) have been used as vaccines against MD over the last four decades [6]. These vaccines generally prevent the development of MDV induced tumors and disease, however, they do not prevent superinfection with pathogenic MDV [7,8]. Thus, at present, it is widely accepted that superinfection of homologous viruses (or live vaccines against MD) have ultimately led to the increasing virulence of pathogenic MDV strains [25].

In addition to homologous superinfection, MDV is often a mixed infection with heterologous viruses, just as mentioned above, ALV-J, REV, AHEV, and CAV. Though the phenomena of superinfection of MDV with heterologous viruses are often observed in nature, the direct reason for the enhanced pathogenicity, tumorigenesis, and expended tumor spectrum still remained unclear.

In the present study, we demonstrated a synergism in viral replication of heterologous superinfection of MDV and ALV-J in vitro and in vivo. In viral synergistic interactions, biological traits such as virus replication, cytopathic changes, tissue tropism, host range, and transmission rates of one or both of the viruses are changed [26,27]. Co-infected viruses may interact directly by complementation of defective functions or indirectly, through host responses such as the defense mechanism [28,29,30]. Initially, single viral infection was thought to be fully responsible for the tumourigenesis of each virus, while now it is established that in many cases, two or multiple viruses collaborate as co-factors in tumor formation and tumor spectrum extending, such as the Kaposi’s sarcoma herpesvirus (KSHV), Epstein Barr virus (EBV), human immunodeficiency virus type 1 (HIV-1), human hepatitis C virus (HCV) showing association with non-Hodgkin’s lymphomas [31]. Superinfection with any one or multiple oncogenic viruses may establish an environment that enhances tumor initiation and progression [32,33]. Thus, enhanced tumorigenesis and pathogenicity are usually observed in host with a superinfection of tumor viruses.

To verify the hypothesis that synergistic viral replication of MDV and ALV-J is the direct cause of the enhanced tumorigenesis and pathogenicity, we tested the CPE in vitro and tumorigenesis and pathogenicity in vivo in MDV and ALV-J dual-infected CEFs and chickens. In vitro, we found that the RNA level and protein expression of MDV and ALV-J in dual-infected CEFs were significantly higher than those of single infection, suggesting that the synergistic viral replication occurs in an appropriate environment. CLSM observation showed that the more viral replication, the more severe CPE was observed in dual-infected CEFs. During the in vivo experiment, we also found that the more viral loads, the more severe tumorigenesis and pathogenicity were present in tissues of dual-infected chickens. Interestingly, in dual-infected spleens, ALV-J drove MDV copies up 889.61-fold, indicating the significant synergism of ALV-J and MDV in immune organs [34].

In addition to the clinical phenotype, experimental models of superinfection have identified a variety of mechanisms that might contribute to tumourigenesis and pathogenicity, including microenvironmental abnormalities [31], viral cofactors [35,36,37], common signaling pathway targets [37,38], epigenetic modifications [39,40,41,42], and interference with cell death [43,44]. The role of cytokines as effectors or predisposing elements in synergistic infections has received prominent attention [45]. Thus, our study is concerned with the IL-6, IL-10, and TGF-β cytokine secretion profile in MDV and ALV-J dual-infected hosts. In agreement with previous studies [46,47], dual-infected cells were found to have significantly higher levels of IL-6, IL-10, and TGF-β at different time points. These cytokines play an important role in the pathogenesis of MDV and ALV-J [48]. Increased IL-10 might partly explain its pathogenic role in immunosuppression. Gurung [49] revealed a novel TGF-β Treg subset in chickens that is activated during MDV infection and tumor formation.

Our findings indicated that MDV and ALV-J superinfection increase disease severity by increasing viral copies. Although these findings contribute to our understanding of the direct synergistic pathogenic mechanisms of MDV and ALV-J, the molecular mechanism by which superinfection promotes host gene expression or signal pathway remains unclear.

## 5. Conclusions

Our findings elucidated that synergistic viral replication of MDV and ALV-J is the direct cause of enhanced tumorigenesis and pathogenicity in dual-infected hosts. We also found that cytokines are involved in the synergistic viral replication of MDV and ALV-J, including IL-6, IL-10, and TGF-β. However, the complex mechanisms underlying the synergism of MDV and ALV-J remain unclear and require further study.

## Figures and Tables

**Table 1:** Primer used for real time PCR.

Target Gene	Orientation	Sequence	Size (bp)
*Meq*	Forward	5′-GTTTCTCCAGATTCCACCTC-3′	231
	Reverse	5′-TGCAACAATGCGTTCTTAT-3′	
*Env*	Forward	5′-TGCGTGCGTGGTTATTATTTC-3′	144
	Reverse	5′-AATGGTGAGGTCGCTGACTGT-3′	
*GADPH*	Forward	5′-GAACATCATCCCAGCGTCCA-3′	132
	Reverse	5′-CGGCAGGTCAGGTCAACAAC-3′	
